# Gamma irradiation-induced genetic variability and its effects on the phenotypic and agronomic traits of groundnut (*Arachis hypogaea*L*.*)

**DOI:** 10.3389/fgene.2023.1124632

**Published:** 2023-04-26

**Authors:** Imane Saibari, Saïd Barrijal, Mohammed Mouhib, Najlae Belkadi, Ahlam Hamim

**Affiliations:** ^1^ Biotechnological Valorization of MicroorganismsLaboratory, Department of Life Sciences, Faculty of Sciences and Techniques, AbdelmalekEssaadi University, Tangier, Morocco; ^2^ Unity of Research On NuclearTechniques, National Institute For Agricultural Research, Tangier, Morocco

**Keywords:** *Arachis hypogaea* L., gamma irradiation, LD_50_, radio-sensitivity, genetic variability

## Abstract

In order to increase genetic variability for the improvement of groundnut, two varieties, namely Kp29 and Fleur11, were treated with six different gamma irradiation doses. A significant effect of mutagenesis was distinctly observed in the stem lengths, roots, and survival percentage in both varieties. The radio-sensitivity test showed a mean lethal dose of 436.51Gy for Kp29 and 501.18 Gy for Fleur11. Furthermore, this study revealed putative mutants with variable agro-morphological traits. Seven chlorophyll mutants and various seed shape and color mutants were obtained. This study demonstrates the potency of gamma irradiation to induce high genetic variability that led to the emergence of certain mutations of economic importance.

## 1 Introduction

Groundnut (*Arachis hypogaea* L.) is a multipurpose legume crop used in both human and animal nutrition due to its high protein (25%–30%) and oil (42%–52%) contents. It is considered one of the major oilseed crops; its worldwide production is 50.35 million metric tons (USDA-FAS, 2022). Groundnut is cultivated in tropical, subtropical, and temperate zones. The use of legumes as groundnut grown in rotations or intercropping is now considered as an alternative and sustainable way of introducing nitrogen into lower input agrosystems (Singh et al., 2021), in which has a high nitrogen fixing ability estimated to be 100–190 kg N.ha^-1^.

In Africa, smallholders consume it in the form of a vegetable, sauce, or artisanal oil. Groundnut oil is also industrially extracted for local consumption or export. It is valuable due to its nutritional qualities, stability, and good behavior in heat. In Morocco, the main groundnut production basin, particularly in irrigated cultivation, is located in the south-east of the Tangier region, particularly in the sandy coastal zone bordering the Atlantic extending between Larache and Kenitra, on approximately 25,000 hectares (Abbou et al., 2022) and its production is 38,000 tons according to statistics from the International Production Assessment Division of USDA’S (USDA-IPAD, 2022). However, this production is subjected to various types of constraints: biotic stresses, such as abundance and diversity of weeds and fungal and microbial infection, and abiotic stresses, such as drought, salinity, and low soil fertility, which affect negatively seedling, vegetative and reproductive growth, seed quality, and yield (Nithila et al., 2013; Verma et al., 2013; Valenzuela-Ruiz et al., 2018).

Groundnut is an allotetraploid species that has very low genetic variability. Therefore, enrichment of genetic diversity through breeding programs is required to increase groundnut yield. Many useful genetic modifications that use different physical and chemical mutagen treatments have been successfully reported for many crops including groundnut (Tshilenge-Lukanda et al., 2013; Hanafy and Akladious, 2018; Nurmansyah et al., 2020). The induced mutation has resulted in the official release of over 3,402 improved mutant varieties in more than 240 species from different crops (Mutant Variety Database (MVD, 2022), of which approximately 70% of these mutants were developed using physical techniques. While these techniques are considered safe and effective, they usually cause large mutations or large-scale deletions of DNA, especially the most widely used gamma ray irradiation (Bado et al., 2015; Oladosu et al., 2016; Spencer-Lopes et al., 2018). Thus, optimization of the radiation dose is the first step in mutation induction, besides the ideal dose depending on the plant materials and desired outcome (Oladosu et al., 2016). The present study aimed to determine the mean lethal dose (LD_50_) of gamma irradiation on groundnut cultivated in Morocco (*Arachis hypogaea* L. var. Kp29 and var. Fleur11) to obtain the genetic diversity of the M2 generation and screen and select mutant plants based on phenotypic and agronomic traits.

## 2 Materials and methods

### 2.1 Plant materials

Two groundnut seed subspecies with erect habit and short cycle of 90–110 days that are widely grown in Northwest Morocco were used: *fastigiata,* variety Kp29 (Valencia type, having three to four seeds per pod); and subspecies *hypogaea*, variety Fleur11(Spanish type, having 2 seeds per pod). Healthy dry seeds of the same size were used. The moisture content of the seeds was kept at 10%–12% until mutagen treatment according to the method described by Spencer-Lopes et al. (2018).

### 2.2 Gamma irradiation treatment

Various doses ofcobalt-60(^60^Co) gamma ray were used to induce mutagenesis in groundnut seeds. Irradiation was performed with a dry storage source of ^60^Cin the Boukhalef Ionisation Station (SIBO) at the Regional Center of Agronomic Research of Tangier. The dose rate was approximately 1.44 Gy/min. Three replicates of 70gofseeds per genotype were placed in 15 × 22 cm zip-lock plastic bags, each containing approximately 80 groundnut seeds. The bags were irradiated with gammarayat0, 100, 150, 200, 350, 600, and 1,200 Gy, with 0 (non-irradiated bag) as the control.

### 2.3 Radio-sensitivity test

Uniform seeds of each variety were surface sterilized with 0.1% HgCl_2_ for 3 min, followed by thorough washing with distilled water. Three replicates of 20 seeds were placed on the sterile Joseph paper in the Petri dish, moistened with distilled water, and kept in the incubator at 28°C for 15 d. Germination was achieved when the radical length of the seeds reached 5 mm and the germination percentage was estimated by counting the number of seedlings at 15 d. After germination, we sowed the seeds in sandy soil and placed them in a greenhouse at 25°C for 30 d. The survival percentage was estimated by counting the viable seedlings 30 d after germination. The lengths of the plant shoot and root were measured using a ruler. These variables are regarded as suitable indicators for estimating the damage caused by mutagenic treatments.

To determine the optimal dose, the LD_50_ value of gamma irradiation on M1 seeds was determined using Probit analysis based on the survival percentage (Postelnicu, 2011). The percentage of germination and survival were calculated using the following formula:

$$Germination\;\left(\%\right)=\frac{No.\;ofgerminatedseeds}{TotalNo.\;ofseeds\;planted}\times 100$$

(Olasupo et al., 2016).

$$Survival\;\left(\%\right)=\frac{No.\;ofsurvivedseedlings}{No.\;ofgerminatedseeds}\times 100$$

(Olasupo et al., 2016).

### 2.4 Field experiments

The experiments were carried out at the experimental station of the National Institute for Agronomic Research in Larache, during the growing season of 2018–2019. The soil of the station consisted of 80.5% sand, 11% silt, and 8.5% clay, with a pH of 5.5-6 and electrical conductivity of 40 mS/m (±25%). Conventional agricultural practices were employed in these experiments. Irrigation was carried out thrice per week by the drip system. M0 and M1 seeds (n = 210) of each dose (0.100, 150, 200, 350, 600, and 1200Gy) of the two varieties (Fleur11 and Kp29) were sown in May 2018. Plots with dimensions of 2.5 × 4.0 m in a randomized complete block design were used. Then, the M1 plants from each irradiation dose were individually harvested and sown as M2 population plots in the next season (May 2019). Groundnut M2 seeds from the two varieties were grown in the field according to the pedigree method, which consists in sowing all seeds from a single plant in a raw (plant to row) (Breseghello and Coelho, 2013). The plots were 10 × 15 m in size, with 11 rows with 50 cm in between rows and 25 cm in between plants. The M0 plants were planted in the first row of each plot under the same culture conditions mentioned above. After sowing, M2 seedlings (600, 400, and 400 seedlings for 100, 200, and 350 Gy, respectively) were screened every week from weeks 4–8 to record the presence of chlorophyll mutants based on the nomenclature reported by (Gustafsson, 1940). The frequency of chlorophyll mutants was calculated using the following formula:

$$Mutation\;frequency\;\left(\%\right)=\frac{NO.\;ofmutants}{TotalNO.\;ofM2\;seedlings}\times 100$$

(Konzak et al., 1965; FAO/IAEA, 2018).

### 2.5 Statistical analysis

Experimental data were statistically analyzed for the analysis of variance (ANOVA), and the means were compared using Tukey’s HSD (Honestly Significant Difference) test. Statistical analyses were performed on IBM SPSS Statistics version 26.0 (IBM Corp, 2019), with statistical significance set at *p* ≤ 0.05.

## 3 Results

### 3.1 Radio-sensitivity test

#### 3.1.1 Germination percentage

The data presented in Table 1 shows that the increasing the dose of gamma rays had no significant effect on seed germination in both varieties. Irradiated groundnut seeds maintained their germination capacity compared with the control. The germination percentage of M1 seeds decreased at 350 and 600 Gy for the Kp29 variety and at 600 Gy for the Fleur11 variety. No significant differences were observed in the survival percentage of M1 and M0 seeds (Table 1).

**TABLE 1:** Germination percentage in groundnut varieties on response to gamma irradiation.

Dose (Gy)	Varieties (%)	
	Kp29	Fleur11
Control	100	100
100	97	98
150	98	97
200	93	97
350	92	95
600	92	90
1,200	95	95
Significance	-	-
(Tukey HSD *p* < 0,05)	Ns	Ns

### 3.2 Survival percentage

**FIGURE 1:**
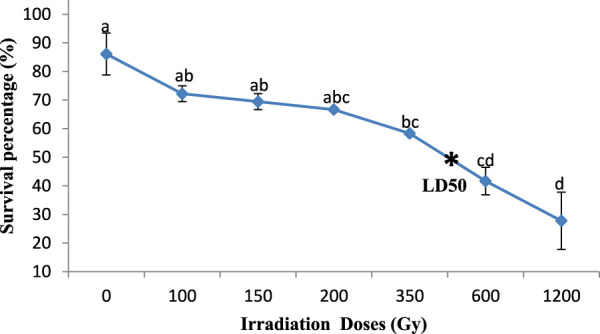
Survival percentage of groundnut var. Kp29 seedlings in response to gamma irradiation. Means with different letters **,**(a,b,c,d) are significantly different (Tukey’s HSD *p* < 0.05).

**FIGURE 2:**
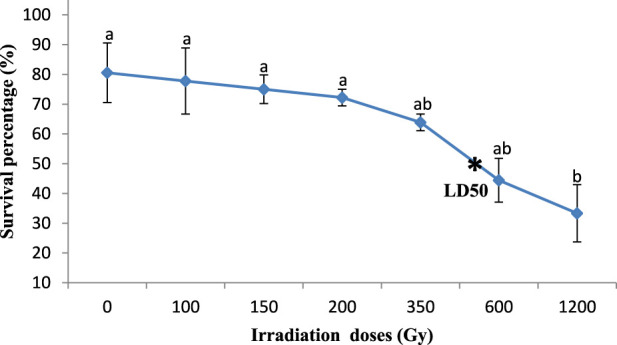
Survival percentage of groundnut var. Fleur11 seedlings in response to gamma irradiation. Means with different letters (a,b,c,d) are significantly different (Tukey’s HSD *p* < 0.05).

Significant differences were observed in the effect of gamma irradiation on the survival percentage of M1 seeds (350, 600, and 1,200 Gy) in the two varieties compared with M0 seeds, reducing their survival percentage from 81.6% and 86.1%– 27.8% and 33.3% (Kp29 and Fleur11, respectively; Figures 1, 2). However, no significant differences were observed in the survival percentage between M1 seeds var. Fleur11 (100, 150, and 200Gy) and M0 seeds (Figure 2). The Probit analysis showed that the LD_50_ was 436.51 Gy for Kp29 and 501.18 Gy for Fleur11 (Figures 1, 2).

### 3.3 Root and shoot length

**FIGURE 3:**
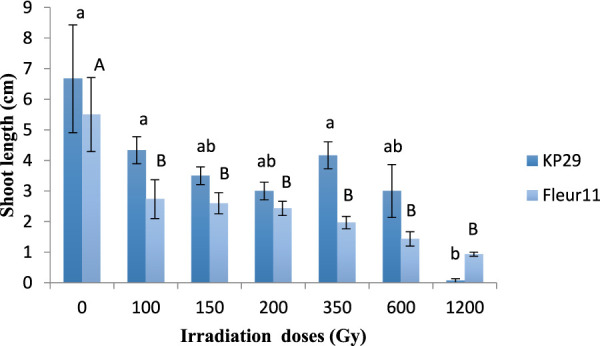
Shoot length of groundnut seedling var. Fleur11 (indicated in uppercase letters) and Kp29 (indicated in lowercase letters). Different letters indicate a significant difference (Tukey’s HSD *p* < 0.05).

A significant effect was observed for shoot and root length besides both varieties. The gamma rays significantly decreased the shoot length. For Kp29, three groups have been established: the first group contains 3 doses 150, 200, 600Gy, the second group with 1,200 Gy with a value of 0.06cm, and the third group including the control, 100Gy and 350 Gy, with values from 6.7 to 4.16 cm, respectively (Figure 3). In the Fleur11 variety, only two groups have been established: the first group of the control with a maximum value of 5.5 cm, and the second group containing the seeds irradiated with doses from100Gy to 1200Gy with values ranging from 2.7 to 0.93 cm (Figure 3).

**FIGURE 4:**
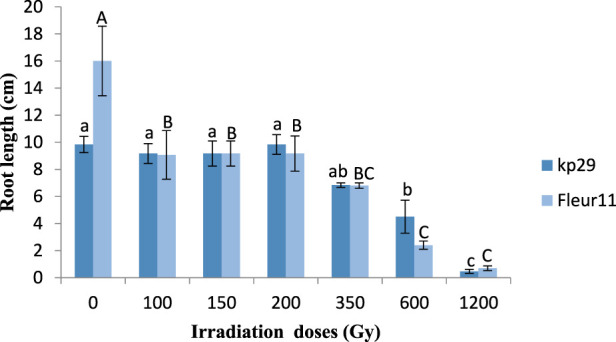
Root length of groundnut seedling var. Fleur11 (indicated in uppercase letters) and Kp29 (indicated in lowercase letters). Different letters indicate a significant difference (Tukey’s HSD *p* < 0.05).

**FIGURE 5:**
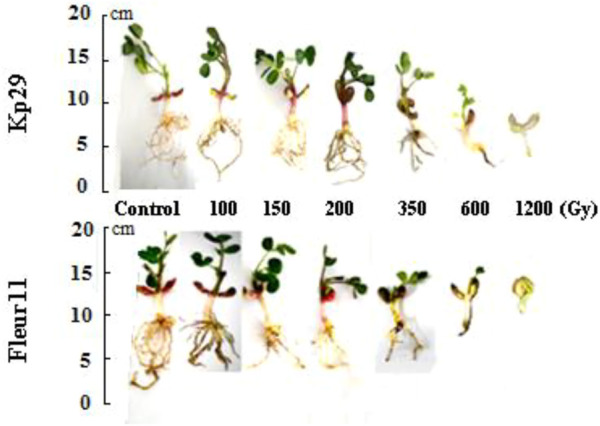
Dose effect of gamma irradiation on shoot and root lengths of two varieties 30 days after germination.

Significant results were observed for the root length of Kp29, wherein seeds irradiated at 100 Gy (16 cm), 150 Gy (9.16 cm), 200 Gy (9.16 cm), 350 Gy (6.8 cm), 600 Gy (2.4 cm), and 1,200 Gy (0.7 cm) showed reduced values when compared to M0 (9.6 cm; Figures 4, 5). Similar results were observed for root length in the Fleur11 variety, with the control having the highest value (16 cm), followed by seeds irradiated at doses of 100 Gy (9.16 cm), 150 Gy (9.16 cm), 200 Gy (10.5 cm), 350 Gy (6.8 cm), 600 Gy (2.4 cm), and 1,200 Gy (0.7 cm). Thus, the greatest negative effect on the root and shoot of groundnut seedlings was observed in seeds irradiated at 1,200 Gy (Figures 4, 5).

### 3.4 M1 generation field experiment

#### 3.4.1 Germination percentage

**FIGURE 6:**
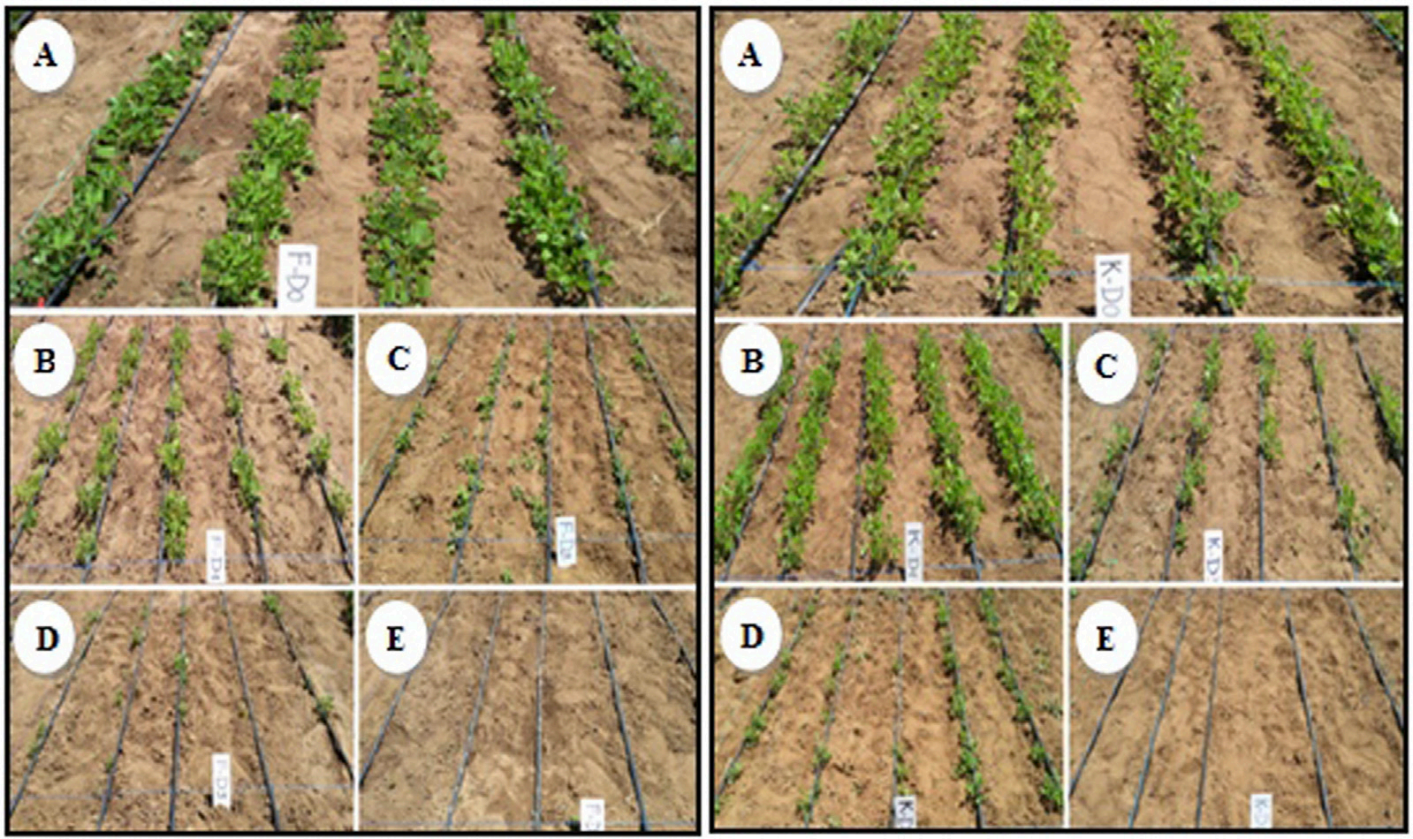
Germination percentage of M1 of groundnut var. Under field conditions, 15 days after sowing. **(A)** Control seeds, **(B)** seeds irradiated at 100 Gy, **(C)** seeds irradiated at 150 Gy, and **(D)** seeds irradiated at 200 Gy, **(E)** seeds irradiated at 350 Gy (left var. Kp29, right var. Fleur11).

In the field, the lowest germination percentage of M1 seeds for Kp29 was observed in those irradiated at 350 Gy (43.3%), which was significantly different compared with the other doses. Compared with M0, seed germination was not significantly affected at 100 Gy (84.3%), 150 Gy (85.2%), and 200Gy (63.8%); however, a decreasing trend was observed (Figure 6; Table 2).

**TABLE 2:** Effect of gamma irradiation on different M2 generation groundnut varieties Kp29 and Fleur11 traits.

Variety	Dose (Gy)	Germination (%)	Survival (%)	Days to flower initiation	Plant height (cm)	Number of pods
Kp29	Control	89.6^a^	89.5^a^	33^b^	51.56^a^	46.3^a^
	100	84.3^a^	74.73^a^	34^b^	47.56^a^	38^ab^
	150	85.2^a^	63.3^a^	43^a^	45.78^b^	34.1^b^
	200	63.8^ab^	62.8^a^	45^a^	40.44^b^	28.8^b^
	350	43.3^b^	5.2^b^	-	-	-
Fleur11	Control	83.3^a^	83.3^a^	34^b^	49.8^a^	42.3^a^
	100	80^a^	80^a^	37^b^	42.1^b^	35.5^b^
	150	64.3^ab^	60.9^ab^	44^a^	39.1^b^	32.6^bc^
	200	55.7^b^	52.8^b^	46^a^	35.3^c^	27^c^
	350	26.2^c^	1.4^c^	-	-	-

Means with a different letters (a,b,c,d) are significantly different (Tukey’s HSD *p* < 0.05).

Similarly, for the Fleur11 variety, the lowest germination percentage was observed at 350 Gy (26.2%), which was also significantly different compared with the other doses. Compared with M0, seed germination was not significantly affected at 100 Gy (80%) and 150 Gy (64.3%; Figure 6; Table2).

#### 3.4.2 Survival percentage

For Kp29, the survival percentage of M1 seeds irradiated at 350 Gy (5.2%) was significantly different compared with the other treatments. Similar to the seed germination percentage, the survival percentage of seedlings was not significantly affected at 100, 150, and 200 Gy compared with M0; however, a decreasing trend was observed for this parameter.

For Fleur11, significant differences in the survival percentage of M1 seeds were observed between 200 Gy (52.8%) and 350 Gy (1.4%), as shown in Table 2.

#### 3.4.3 Days to flower initiation

Flower initiation was significantly delayed in plants of both varieties for all irradiation doses compared with M0 plants. Compared with M0 plants (33 d), the longest delay was observed for Kp29 plants irradiated at 200Gy (45 d), followed by 150 Gy (43 d), and 100 Gy (34 d). For Fleur11, the longer delay was observed at 200 Gy (46 d), followed by 150 Gy (44 d) and 100 Gy (37 d) when compared with M0 plants (34 d; Table2). Meanwhile, all seeds irradiated at 350Gy died a few days after germination, and no results were reported before the flowering stage.

#### 3.4.4 Plant height

Significant differences in plant height were observed among different irradiation doses. For the Kp29 cultivar, the lowest heights were observed at 200 Gy (40.44 cm), 150 Gy (45.78 cm), and 100 Gy (47.56 cm) compared with M0 (51.56 cm; Table 2). For the Fleur11 cultivar, the lowest heights were observed at 200 Gy (35.3 cm), 150 Gy (39.1 cm), and 100 Gy (42.1 cm) compared with M0 (49.8 cm; Table 2).

#### 3.4.5 Number of pods

Similar results as the indicators mentioned above were observed for the number of pods. The number of pods in the two varieties was significantly reduced, with the highest number being observed for non-irradiated plants (46.3 in var. Kp29 and 42.3 in var. Fleur11) and the lowest being at dose 200Gy (28.8 in Kp29 and 27 in Fleur11; Table 2).

### 3.5 M2 generation field experiment

#### 3.5.1 Chlorophyll mutants

**FIGURE 7:**
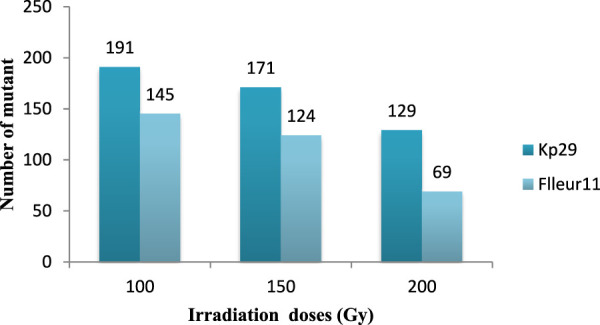
Total numbers of mutants selected from different doses of gamma irradiation in M2 generation.

**FIGURE 8:**
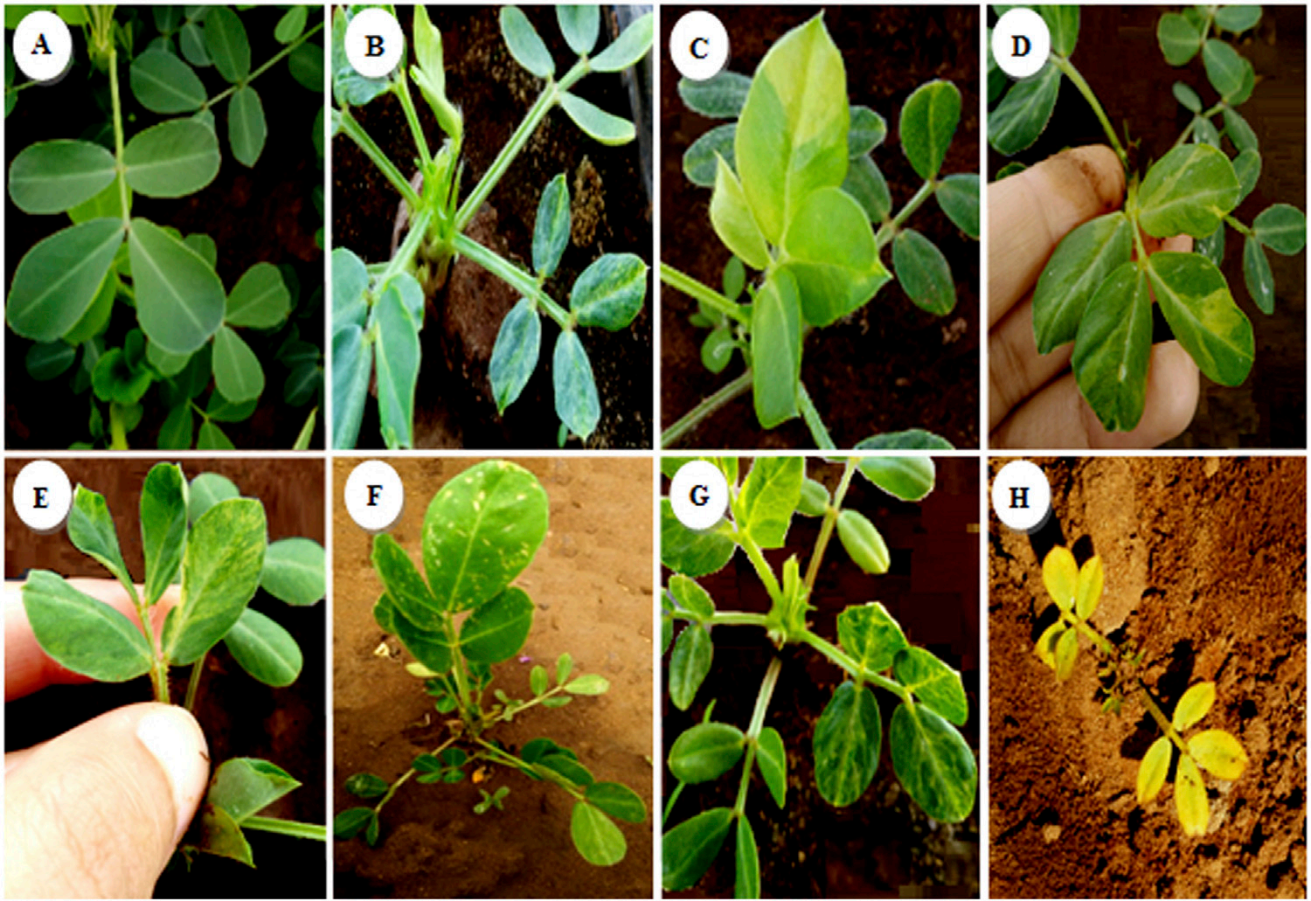
Spectrum of chlorophyll mutants in the M2 generation of groundnut irradiated. **(A)** Control, **(B)** variagated, **(C)** Yellow-viridis, **(D)** Striata, **(E)** Viridis, **(F)** Maculata, **(G)** Chlorina, **(H)** Xhanta.

Seven different types of chlorophyll appeared in the segregating generation: variegated mutants (Figure 7B) typically had green- and yellow-sectored leaves; yellow-viridis mutants (Figure 7C) had yellow green leaves; striata mutants (Figure 8D) showed longitudinal yellow stripes; viridis mutants (Figure 8E) had viridine green-colored leaves; maculata mutants (Figure 8F) exhibited chlorophyll and carotin destruction in the form of dots over the leaves; chlorina mutants (Figure 8G) had leaves that were yellowish to light green in color and survived within 20 days and xhanta mutants (Figure 8H) were completely yellow and could survive a few days after germination. All of the chlorophyll mutants types except striata, variagated, and viridis mutants were lethal and only survived to seedling stage.

Different types of chlorophyll mutants were observed in the M2 generation of both varieties at different doses. The highest frequencies of chlorophyll mutants (6.15% var. Kp29% and 4.10% for var. Fleur11) were observed at 200 Gy, and the lowest frequency was recorded at 100 Gy (0.22% in both varieties; Table 3).

**TABLE 3:** Frequency and number of chlorophyll mutants types in M2 generation of groundnut in two varieties studied.

	Kp29			Total of chlorophyll types	Fleur11			Total of chlorophyll types
Number of M2 seedling	100Gy	150Gy	200Gy		100Gy	150Gy	200Gy	
	452	289	195		446	335	268	
Variagated (B)	0	0	2	2	0	1	2	3
Yellow viridis (C)	0	1	2	3	0	2	3	5
Striata (D)	1	1	2	4	1	1	2	4
Viridis (E)	0	1	2	3	0	1	1	2
Maculata (F)	0	0	1	1	0	1	1	2
Chlorina (G)	0	1	2	3	0	1	1	2
Xhanta (H)	0	0	1	1	0	1	1	2
Total	1	4	12	17	1	8	11	20
Frequency (%)	0.22	1.38	6.15	7.25	0,22	2.38	4.10	6.70

#### 3.5.2. Phenotypic and agronomic characteristics of mutants

**FIGURE 9:**
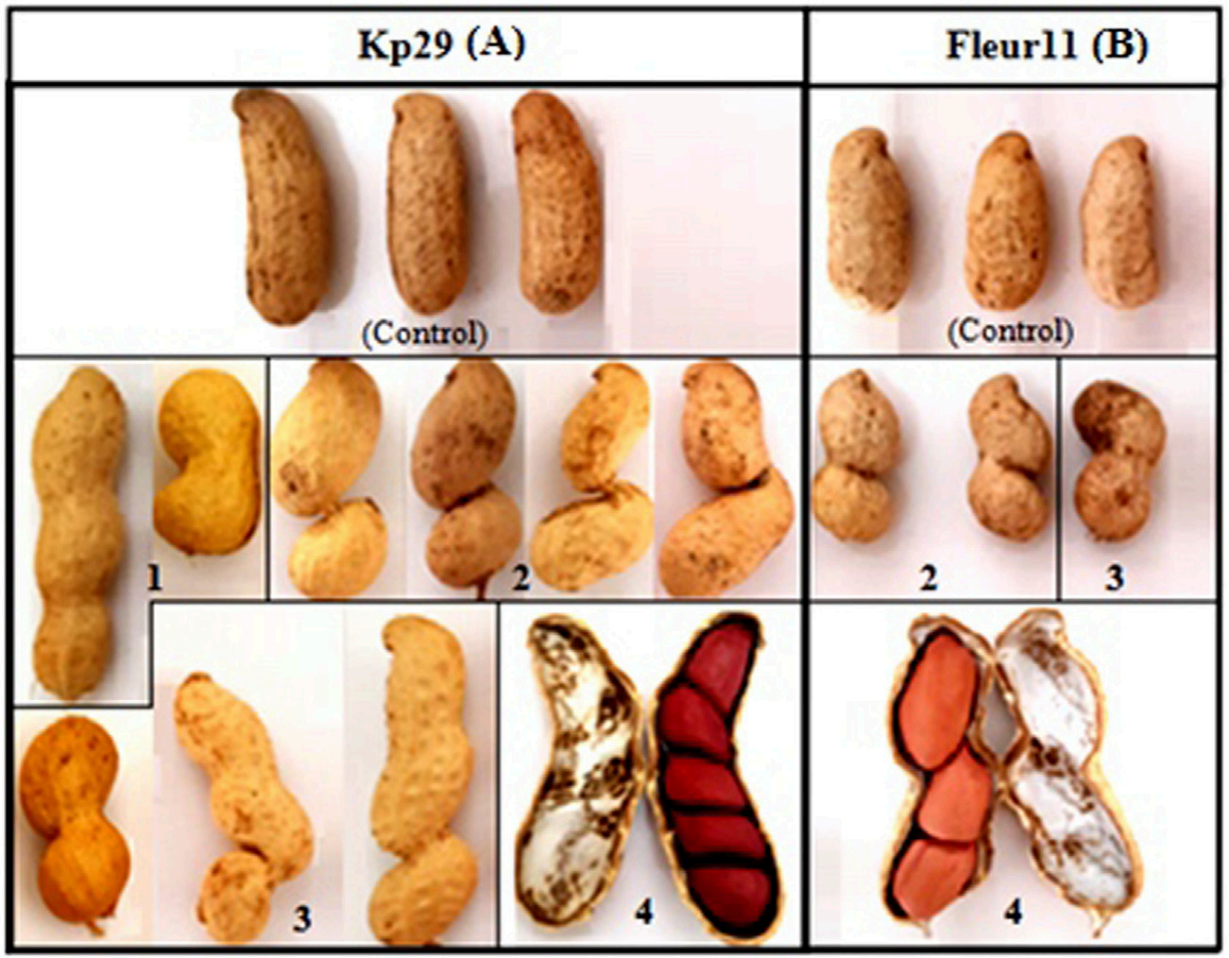
Pods shape variation of groundnut mutants in both varieties, **(A)** Kp 29, **(B)** Fleur 11. **(1)**: slight, **(2)**: very deep, **(3)**: deep, **(4)**: number of seeds per pod.

The mutant number varied according to the irradiation dose and variety. The highest number of mutants was observed at 100 Gy (191 for Fleur11 and 145 mutants for Kp29; Figure 9). The frequencies of selected mutants were clustered into different groups to measure the extent of variability achieved through irradiation as shown in Table 4.

**TABLE 4:** Frequency of phenotypic characters of the selected mutants in M2 generation of groundnut var. Fleur11 and var. Kp29.

Relative frequency of agronomic mutants (%)	Kp29				Fleu11			
	100Gy	150Gy	200Gy	100Gy		150Gy	200Gy	
Early flowering	19.02	18.68	15.38	8.29		6.56	6.71	
Shape mutant seeds	25.2	33.6	40.1	4.9		13.8	17.8	
Color mutant coat seeds	8.7	9.7	6.4	24.7		13.1	9.5	
Color coat/shape mutants seeds	2.8	8.4	6.4	1.7		8.3	2.9	
Number of seed								
per pods								
1–2	16.50	10.61	7.44	100		98.96	99.40	
3–4	83.50	88.93	92.02	0		1.04	0.60	
≥5	0	0.45	0.53	0		0	0	

The yield of the M2 generation was screened, and differences were observed. Plants carrying a putative mutation for early flowering were found: 84 (100 Gy), 54 (150 Gy) and 30 (200 Gy) plants for Kp29; and 37 (100 Gy), 22 (150 Gy), 19 (200 Gy) plants for Fleur 11. Mutations for the shape of pods and seeds were observed: 71 (100 Gy), 75 (150Gy), and 75 (200 Gy) plants for Kp29; and 17 (100Gy), 20 (150Gy), and 30 (200 Gy) plants for Fleur11. Meanwhile, 25 (100 Gy), 25 (150 Gy), and 12 (200 Gy) plants for Kp29, and 85 (100Gy), 11 (150 Gy), and 18 (200Gy) plants for Fleur11 had mutations for seed color.

Mutations for color and shape were observed in 12 (100 Gy), 19 (150 Gy), and 8 plants (200 Gy) for Kp29 and in 5 (100 Gy), 22 (150 Gy), and 7 (200 Gy) plants for Fleur11.

In terms of the number of seeds per pods, 1 (100 Gy) and 1 (200 Gy) plant for Kp29, and 3 (100 Gy) and 1 (200 Gy) plant for Fleur11 showed mutations.

**FIGURE 10:**
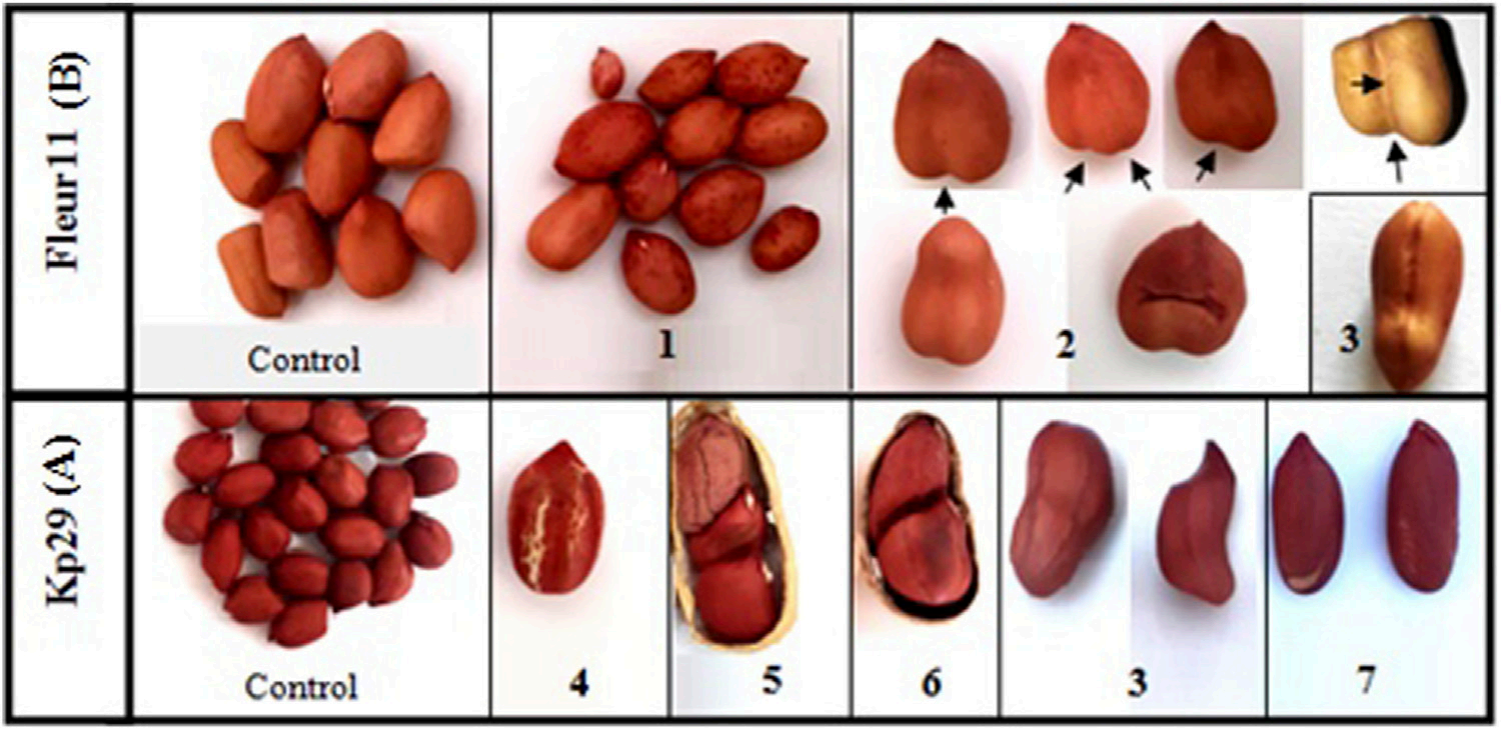
Seed coat color and shape variations of groundnut mutants. 1: red spotted color, 2: seeds with different concavity forms, 3: large mutant, 4: white strips mutant, 5: light red color coat, 6: black spotted color coat, 7: beige spotted color coat. **(A)**: Kp 29, **(B)**: Fleur 11.

The different seed characteristics of mutant plants, including seed coat color, seed shape, and pod shape, are shown in Figures 9, 10. Three different pod shapes were observed: slight (Figure9A1), deep (Figure 9A-B3), and very deep (Figure 9A-B2). Meanwhile, two seed shapes were observed: seeds with different concavity forms and positions and large seed mutants (Figure 10A-B2, 3). A high number of seeds per pod (Figure 10A-B4) was also noted. Furthermore, different seed coat colors were observed: red spotted color (Figure 10A1); white strips (Figure 10A-B4); light-red coat color (Figure 10B5); black spotted coat color (Figure 10B6); and beige spotted coat color (Figure 10B7).

## 4 Discussion

### 4.1 Radiosensibility test

In this study, the radiosensitivity test of two groundnut varieties showed that the germination percentage was not affected by different irradiation doses (Table1). This result is consistent with that of Essel et al. (2016), which showed no significant effect on cowpea seeds in all treatments, suggesting that physical treatment does not have any effect on seed germination. Similarly, Hameed et al. (2008) noted that the germination percentage of chickpea seeds was not significantly affected by gamma irradiation at all of the irradiation doses used. Ahumada-Flores et al. (2021) also reported a delay in germination time of wheat seeds, but no effects were observed on the final germination percentage after irradiation at varying doses. The relatively high germination percentage recorded in this study could be explained by the stimulating effect of irradiation on RNA or protein synthesis (Kuzin et al., 1973).

Meanwhile, based on the survival percentage of seedlings of irradiated seeds, the obtained LD_50_ was 436.51 Gy for var. Kp29 and 501.18 Gy for var. Fleur11. The difference in genotype sensitivity can be explained by the difference in the genotype between genetic patterns as clarified by Khursheed et al. (2015) or in the nature of their seed coat and size (Olasupo et al., 2018). The large orange-beige seeds of Fleur11 had a lower dose compared with Kp29, which was noted for the small red grains. Similar findings have been previously reported in the LD_50_ of groundnut, which was found to be between 400 and 600 Gy (Tshilenge-Lukanda et al., 2013). Meanwhile, Aparna et al. (2013) observed a 50% reduction in all germination parameters of Narayani groundnut species at a radiation dose of 1,500 Gy. Furthermore, Majeed et al. (2018) highlighted that the reduction in the survival of plants is an index of post-germination mortality resulting from cytological and physiological disturbances caused by irradiation.

In addition, the present findings showed a reduction in the mean height of shoot and root in both varieties as the radiation dose increased. A similar study by Gunasekaran and Pavadai (2015) on groundnut showed that the effect of gammaray treatment on growth parameters of M1 generation decreased with increasing doses. In addition, Aparna et al. (2013)reported that gamma radiation resulted in lower values of groundnut. This reduction was also observed in several other species: cowpea (*Vigna unguiculata* [L.] Walp) (Gnankambary et al., 2019), okra (*Abelmoschusesculentus* L. Moench.) (Asare et al., 2017), pea (*Pisumsativum* L.) (Majeed et al., 2016), rice (*Oryza sativa* L.) (Gowthami et al., 2017), and maize (*Zea mays* L.) (Matova et al., 2021). This reduction could be attributed to the inefficiency of plant growth hormone and auxin or to the influence of the ionizing radiation, which causes chromosomal aberration in both mitotic and meiotic cells (Mondal et al., 2017; Nurmansyah et al., 2020). Otherwise, indirect damage of the entire genome may partly be responsible for the dose-dependent induction of oxidative stress and reactive oxygen species (Ford et al., 2018), which react rapidly with other cellular molecules, exerting negative structural effects and inactivating them. These changes can trigger growth abnormalities (Majeed et al., 2018).

### 4.2 M1 generation field experiment

Similar to the laboratory assay, the lowest seedling survival percentage of irradiated groundnut seeds was observed in the field at a higher irradiation dose, 350 Gy, which was significantly different compared with other irradiation doses. Gamma irradiation has been reported to have negative consequences on seedling development due to various damages in the whole genome, such as deletions of DNA nucleotide sequences that may lead to reading-frame shifts, inactive protein products, or faulty transcripts (Manova and Gruszka, 2015).

In addition, the M1 plants from irradiated seeds had a longer period before flowering, whereas those from the control had a shorter period (Table 2). Similar results were observed by Essel et al. (2016) and Verma et al. (2018), reporting that increasing irradiation doses delayed flowering initiation.

Regarding the number of pods, higher irradiation doses resulted in decreased numbers. For the KP29 variety, 46.3 pods were observed for plants from M0 seeds, while 28.8 pods were observed for plants from seeds irradiated at 350 Gy (Table 2). However, Majeed et al. (2016) observed that increasing gamma irradiation doses did not alter the number of pods in pea plants, which contradicts the current findings. Moreover, Smýkal et al. (2012) confirmed that the number of pods depend on several factors, both endogenic and exogenic.

Plant height is generally used as an index to confirm the biological effects of mutagenesis. Groundnut varieties showed a reduction in plant height with increasing doses of gamma radiation. Similar findings have been reported by Ahumada-Flores et al. (2021), increase in the dose reduced plant height in wheat. This reduction could be due to the effects of gamma ray that may have altered the expression of the gibberellic acid 2-oxidase gene that controls the plant height affecting the vegetative growth of plants (Ford et al., 2018).

### 4.3 M2 generation field experiment

Chlorophyll mutation frequency is one of the most reliable phonological markers for evaluating mutagen-induced genetic alterations. Identification of chlorophyll mutants in the M2 generation was based on the intensity of pigmentation at the seedling stage (Swaminathan et al., 1962; Harten, 1998; Wani et al., 2011; Wani et al., 2017). A large number of chlorophyll mutants has been reported in many crops, and this can be attributed to various causes, such as impaired chlorophyll biosynthesis, further degradation of chlorophyll, and bleaching due to carotenoid deficiency (Bevins et al., 1992; Shah et al., 2006). In addition, mutation of this trait is more likely to be heritable in the segregating generations due to their genes being located on several chromosomes; these genes could be adjacent to the centromere and proximal segment of the chromosome (Gustafsson, 1940; Swaminathan et al., 1962; Gaul, 1964; Goud, 1967; Chopra, 2005).

In this study, seven different types of mutants were observed in the M2 generation under different doses of gamma irradiation. Among these doses, the highest number of chlorophyll mutants was recorded at 200 Gy. Consistent with our results, previous studies by Wang et al. (2013) found that significant changes in chlorophyll development always resulted in the variation of leaf color. Additionally, various chlorotic mutants defective in ChlH, ChlD, or ChlI, which encode the Mg-chelatase subunits, have been identified in *Arabidopsis*, rice, barley, and tea (Mochizuki et al., 2001; Jung et al., 2003). Meanwhile, Papenbrock et al. (2000) reported that the loss of chlorophyll is caused by either a reduction or an excess accumulation of ChlI.

Among the types of chlorophyll mutants recorded in the two varieties, yellow-viridis and striata were the most dominant, followed by viridis, variegated, and chlorina. The reason for the appearance of yellow-viridis may be attributed to the involvement of polygenes in chlorophyll formation (Gaul, 1964). A study by Ambarkar et al. (2005) reported the predominance of viridis among chlorophyll mutant types in chickpea. Sjödin, (1962) and Kumari et al. (2019) reported that viridis was the most common type in *Vicia faba* and rice bean, followed by xantha, while albino was a rare mutant as in most Leguminosae. This is inconsistent with the results of Khan et al. (2005), where in the highest number was observed for xhanta mutants in chickpea and Black Gram (Lal et al., 2009).

In addition, we observed that increasing the frequency of chlorophyll mutation depends on increasing irradiation doses. Similar findings that higher doses of mutagens induce a higher frequency of chlorophyll mutations have been previously reported (Wani et al., 2011; Mishra et al., 2013; Patil and Rane, 2015). In contrast, Nadarajan et al. (1982)andWani et al. (2011) reported that chlorophyll mutations are dose independent. A higher frequency of chlorophyll mutants was observed at lower or medium doses of the mutagen in various crops. These behaviors in chlorophyll mutants can be associated with the saturation of mutational events, which may result in the elimination of mutant cells during plant growth as reported by Kozgar (2014), and with the nature and genes involved in each specific chlorophyll mutation (FAO/IAEA, 2018), which remains relatively unclear (Kolar et al., 2011; Li et al., 2013).

Early flowering mutant plants were mostly recorded at 100Gy, followed by 150Gy and 200Gy in both varieties. Similar results were reported by Verma et al. (2018), who observed significant variation in the number of days before flowering in coriander and fennel through induced mutation. This can be attributed to various hormones such as florigen that may have also been induced or inhibited to initiate or impede flowering.

Other types of morphological mutants, including color, shape, and seed number mutants, were observed at all irradiation doses, and the mutation frequency for these traits increased with increasing doses. At irradiation doses of 100 and 150Gy, the highest frequency of morphological mutants, including seed coat color mutants, seed shape mutants, pod mutants, and plants that had either shape/color mutations, was recorded. Similarly, Chen et al. (2020) observed different mutants in various phenotypic traits, including leaf color and seed size and color, induced by ethyl methane sulphonate in groundnut. Seed mutants were also found in different legume crops induced by physical and chemical mutagens. Wani and Anis, (2008) found three mutants in chickpea following gamma radiation exposure and EMS induction, which gave different seed shapes and showed higher production compared to the control seeds. Wani et al. (2017) also reported bold seed and long pod mutants in mungbean, as well as Nurmansyah et al. (2020), Mondal et al. (2017) and Barshile, (2018), evaluated different seed shapes and coat colors in faba bean, groundnut and chickpea.

## 5 Conclusion

This study allowed us to reveal that gamma irradiation can be used to generate novel agronomic and phenotypic variants of groundnut crops in Morocco. The highest number of mutants was identified at 100 and 150 Gy. The variations appear as different morphological characteristics depending on the variety. Furthermore, we determined LD_50_ from the radiosensitivity test, which will benefit future mutagenic research. This genetic gain can be stabilized in segregating generations, and the genome of desirable mutants can be used to develop and increase variability of groundnut.

## Data Availability

The original contributions presented in the study are included in the article/supplementary material, further inquiries can be directed to the corresponding author.
